# A theoretical quest for high temperature superconductivity on the example of low-dimensional carbon structures

**DOI:** 10.1038/s41598-017-16038-5

**Published:** 2017-11-17

**Authors:** C. H. Wong, R. Lortz, E. A. Buntov, R. E. Kasimova, A. F. Zatsepin

**Affiliations:** 1 Institute of Physics and Technology, Ural Federal University, Ekaterinburg, Russia; 2 Department of Physics, The Hong Kong University of Science and Technology, Clear Water Bay, Kowloon, Hong Kong

## Abstract

High temperature superconductivity does not necessarily require correlated electron systems with complex competing or coexisting orders. Instead, it may be achieved in a phonon-mediated classical superconductor having a high Debye temperature and large electronic density of states at the Fermi level in a material with light atoms and strong covalent bonds. Quasi-1D conductors seem promising due to the Van Hove singularities in their electronic density of states. In this sense, quasi-1D carbon structures are good candidates. In thin carbon nanotubes, superconductivity at ~15 K has been reported, and it is likely the strong curvature of the graphene sheet which enhances the electron-phonon coupling. We use an ab-initio approach to optimize superconducting quasi-1D carbon structures. We start by calculating a *T*_c_ of 13.9 K for (4.2) carbon nanotubes (CNT) that agrees well with experiments. Then we reduce the CNT to a ring, open the ring to form chains, optimize bond length and kink structure, and finally form a new type of carbon ring that reaches a *T*_c_ value of 115 K.

## Introduction

With the recent discovery of superconductivity at 203 K in H_2_S under pressure^1^, it has been demonstrated impressively that high temperature superconductivity can be achieved not only in strongly correlated electron systems, but may also be realized by tuning the Debye temperature and the electronic density of states on the Fermi level in materials with light atoms and strong covalent bonds. Quasi-1D carbon structures are good candidates because the one-dimensionality causes van Hove singularities in the electronic density of states, and the recent progress in the fabrication of carbyne^2–4^ offers new opportunities. Indeed, superconductivity at ~15 K has already been reported in thin carbon nanotubes^5^. However, while the van Hove singularities in nanowires, nanotubes or atomic chains in the one dimensional (1D) limit can under certain circumstances cause very high electronic densities of states at the Fermi level, the low dimensionality is highly unfavorable for applications if they become superconducting. The low dimensionality strongly enhances fluctuations in the phase of the superconducting order parameter that causes phase slip events in a 1D structure and a non-zero resistance at any finite temperature^6^. This is well described by the Langer-Ambegaokar-McCumber-Halperin (LAMH) theory^6,7^ and has been demonstrated in numerous experiments^6,7^. However, there is one way out of this dilemma: to arrange the 1D elements to form an array of parallel wires or a network with finite Josephson coupling^8^. In this case, the normal state properties such as the electronic density of states largely preserve their 1D character, while superconducting order parameter fluctuations are strongly suppressed^8^. It is thus essential in our model to arrange our superconducting elements in an array, and not only tune the structure of the elements, but also to optimize the lateral spacing of the carbon elements within an array. Our work serves to predict the optimal carbon structure to achieve high temperature superconductivity theoretically, but leaves the question open on how to realize such an optimized structures experimentally^9^. However, since the discovery of superconductivity in 1911 high temperature superconductivity has only been observed in a comparatively small number of materials^1,10,11^, and therefore our theoretical approach to optimize the electron phonon coupling by tuning structural modifications starting from a known structure may lead to novel approaches to fabricate tailor-made superconductors.

Low-dimensional carbon structures have been realized in the form of graphene^12^, buckyballs^13^, nanobelts^14^, carbon nanotubes^5^, and most recently in the form of single atom chains of carbon atoms (carbyne)^15^. The electron phonon coupling and superconductivity of those carbon structures are still hot research topics^16–19^. The carbyne has attracted considerable interest due to the theoretical prediction of its extremely high tensile strength, Debye temperature and stiffness that breaks all previous records of other known materials so far^15,20^. Apart from this, the high Debye temperature of the carbyne may be able to activate high temperature superconductivity. Its fabrication is extremely difficult, although a recent breakthrough has demonstrated the feasibility^2–4^. Linear carbyne chains have been predicted to be metallic below 500 K with a structure that consists of repeating double bonds (β-carbyne or cumulene), which transforms above 500 K into the semiconducting α-carbyne (polyyne) with alternating single and triple bonds via a Peierls transition^15^. The unusual fact that the metallic phase occurs here at lower temperature provides hope to use such carbyne chains in the future as one-dimensional building blocks in a quasi-one-dimensional bulk single crystalline structure, in which the carbyne chains are arranged in parallel to form an array.

**Figure 1:**
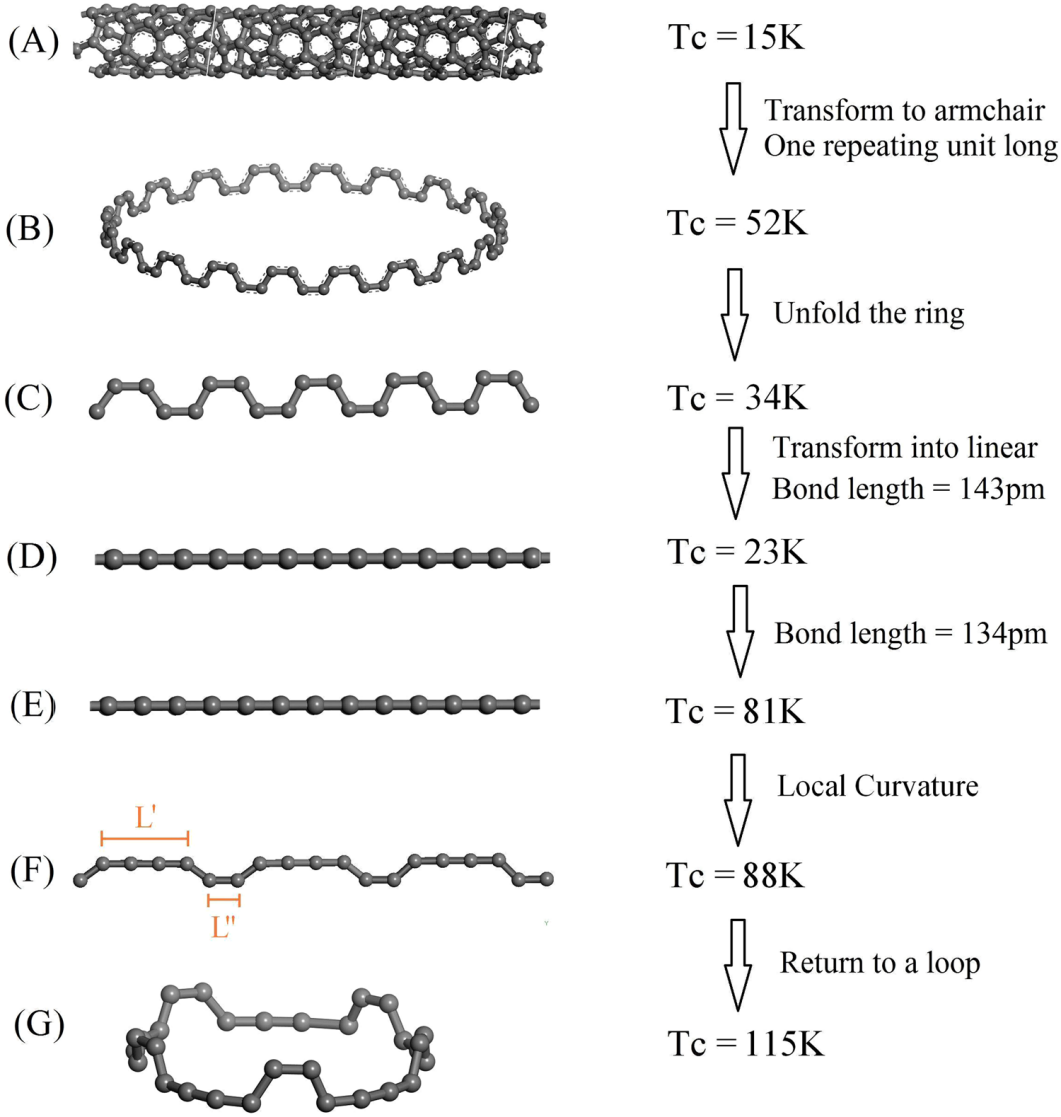
The journey of designing a 115 K superconductor. The various types of carbon materials are shown and labelled as Sample A to G, respectively. Sample A is an infinitely long (4,2) SWCNT (only 3 repeating units are shown). The kink angles of Sample B and C are 60 degree and is reduced to 10 degrees in Sample F. Sample D and E are the infinitely long linear carbon chains with the trivial kink angle of 0 degree. The bond lengths of Sample A, B, C, D are 143 pm. In contrast, the bond distances of Sample E, F, G, are 134 pm. Each sample in the simulation contains about 100 atoms except Sample A, D and F. The ratio of branch length ${R}_{B}=L\text{'}/L\text{'}\text{'}$ in Sample F equals to 3.

We use ab-initio density functional calculations to derive the electronic band diagram, dispersion relationship of phonons and the density of states of electrons and phonons by the GGA functional (CASTEP)^21,22^ in Materials Studio 7. Instead of calculating the superconducting critical temperature directly from Bardeen-Cooper-Schrieffer (BCS) theory, which in case of 1D superconducting materials may become inaccurate, we use a phenomenological scale factor approach. Please refer to the ‘Methods’ section of this paper for all the details. Basically, the final form of the BCS *T*_c_-formula requires a constant DOS as a function of electron energy. However, this is valid only if the Debye temperature is small compared to the range over which the DOS varies significantly. In case of 1D materials this assumption is in many cases not justified, because of the presence of narrow peak-like structures associated with Van Hove singularities in the electronic density of states. Therefore, fundamental assumptions of the BCS *T*_c_-formula become violated. Starting from the known elementary superconducting material represented by arrays of 4 angstrom carbon nanotubes, we gradually modify the structure via a carbon ring, various 1D carbyne structures with and without kinks, towards a kink structured novel type of carbon ring, while optimizing the superconducting transition temperature until a maximum transition temperature of 115 K is found. Figure 1 illustrates our journey towards a phonon mediated high temperature superconductivity in these carbon structures. We investigate how the different parameters, like bond angle or bond length, but also the separation in a bulk structures formed by arrays of such low dimensional elements affect the superconducting transition temperature.

Figure 1 shows the *T*_c_ of the various carbon structures we have investigated. The theoretical *T*_c_ of the (4,2) SWCNT arrays is ~15 K. Here a scale factor derived from the gap and *T*_c_ values of either Al, Ta, Hg, Mo, Ga, Pb, In or Sn in combination with the intermediate step of the sample D was used, as described in detail in the Methods section. It is consistent to the experimental *T*_c_ of the 4 angstrom SWCNT (Sample A) at 15 K^5^ and provides an indirect proof of the curvature-independent scale factor. On the other hand, the theoretical *T*_c_ of the (5,0) SWCNT is ~9 K with help of the scale factors again as shown in Tables 1 and 2. *Rw*, ${R}_{DOS}$,${R}_{kink}$,${R}_{shape}$,${R}_{\omega }$,${R}_{M}$,${R}_{u}$,${R}_{a(q)}$ and$f({T}_{D})$are defined in the Methods section. By comparing Tables 1 and 2, modifying${g}_{kk\text{'}}$ is accurate enough to provide a good reliability when considering the effect of weak curvature and dimensionality in the carbon nanotubes^23^.

**Table 1:** The *T*_c_ values of SWCNT by the modification of ${g}_{kk\text{'}}$ and surface phonon softening^5^.

Material	*R*_*W*_	*R*_*DOS*_	*R*_*Shape*_	*R*_*kink*_	*λ*_*scale*_	µ	*f*(*T*_*D*_)	${T}_{c}^{scale}$ (K)	${T}_{c}^{ex}$ (K)
(4,2)SWCNT	3.9	0.13	1.69	0.85	3.82	0.14	1.0062	16.3	15.0
(5,0)SWCNT	4.3	0.08	1.20	0.85	1.85	0.13	1.0157	7.6	7–15

**Table 2:** The *T*_c_ calculations of SWCNT based on the original Hamiltonian^5^.

Material	***R***_*W*_	*R*_*DOS*_	${{\boldsymbol{R}}}_{\omega }$	${{\boldsymbol{R}}}_{M}$	${{\boldsymbol{R}}}_{u}$	${{\boldsymbol{R}}}_{a(q)}$	${{\boldsymbol{\lambda }}}_{scale}$	µ	${\boldsymbol{f}}({{\boldsymbol{T}}}_{D})$	${T}_{c}^{scale}$ (K)
(4,2) SWCNT	3.9	0.13	1.7	1	1.8	0.98	3.64	0.14	~1	13.9
(5,0) SWCNT	4.3	0.08	1.6	1	1.6	0.99	2.29	0.13	~1	9.5

The (4,2) and (5,0) SWCNT with the Debye temperature of 1000 K are used. We compare the accuracy between the McMillian method and scale factor approach in the 1D regime. We estimate the electron phonon scattering term of the (4,2) and (5,0) SWCNT from ${\lambda }_{Mc}=2\int \frac{{\alpha }^{2}(\omega )F(\omega )}{\omega }d\omega =\frac{DOS({E}_{F})\langle {g}^{2}\rangle }{M\langle {\omega }^{2}\rangle }$, respectively, where $\langle {g}^{2}\rangle$ is the average over the Fermi surface of the square of the electronic matrix element and *M* is the mass of atom^24^. However, the ${\lambda }_{Mc}$values are much smaller than the ${\lambda }_{scale}$values (see Tables 2 & 3). The margin between the ${\lambda }_{scale}$ and ${\lambda }_{Mc}$values is likely increasing when going from the 3D to the 1D regime. The reason is also found in the van Hove singularities in ${\lambda }_{Mc}=\frac{DOS({E}_{F})\langle {g}^{2}\rangle }{M\langle {\omega }^{2}\rangle }$. Even worse, there remain significant uncertainties in values of ${\lambda }_{Mc}$ obtained in this way, which are caused by the presence of the transmission coefficients of tunneling and by inelastic effects across the barrier region^25^. In addition, the McMillian method fails to predict the *T*_*c*_ of SWCNT because the Van Hove Singularities in the electronic DOS are not taken into consideration. Therefore it is wiser to calculate the electron phonon coupling of 1D materials by the original form of the Hamiltonian. The α of SWCNT is obtained by resolving the repeating unit into orthogonal directions, as described in Method section.

**Table 3:** The comparison between the *T*_*c*_ of the SWCNT obtained from the McMillian *T*_*c*_ formula and experimental data^5^.

SWCNT	${{\boldsymbol{\lambda }}}_{Mc}={\bf{2}}\int {{\boldsymbol{\alpha }}}^{{\bf{2}}}({\boldsymbol{F}}({\boldsymbol{\omega }})/{\boldsymbol{\omega }})d{\boldsymbol{\omega }}$	µ	${T}_{c}^{Mc}$ *(K)*	${T}_{c}^{ex}$ *(K)*
(4,2)	0.62	0.14	6.1	15
(5,0)	0.31	0.13	0.2	7–15

**Figure 2:**
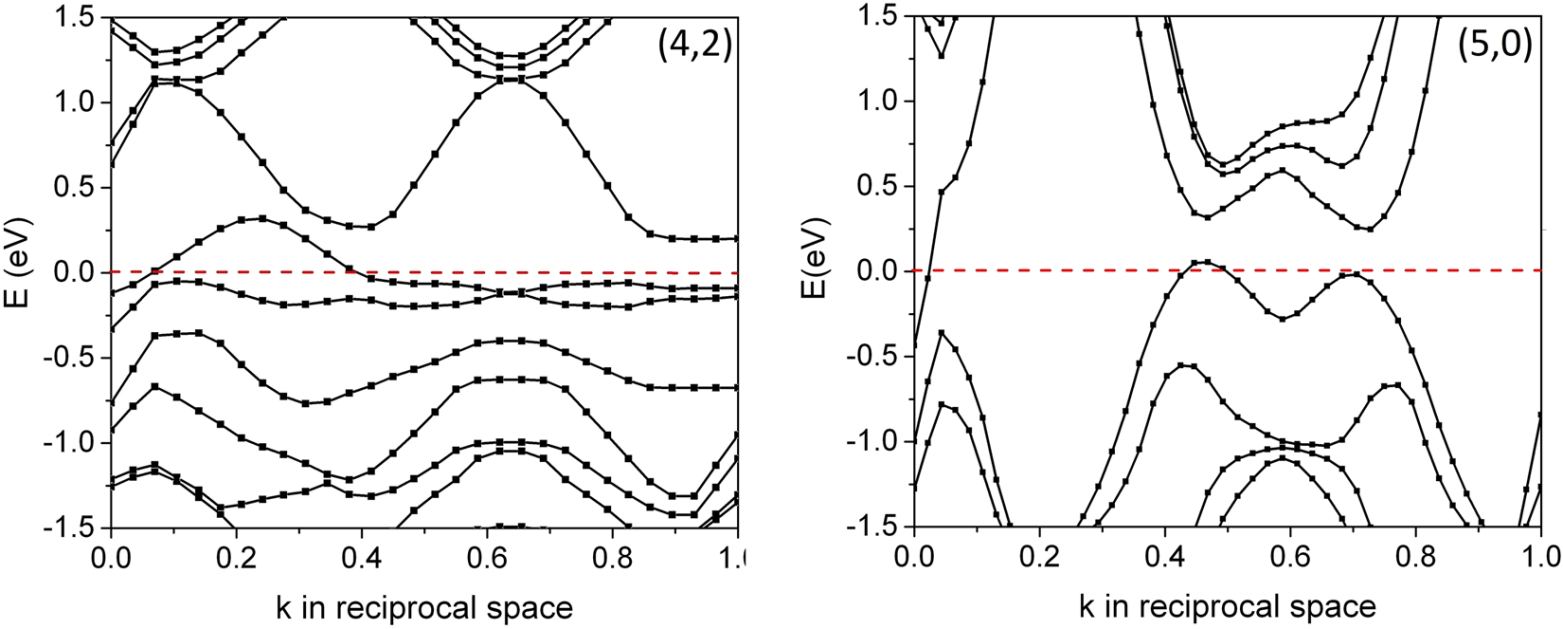
The energy band diagrams of the hexagonal arrays of (4,2) and (5,0) SWCNT. Both show a metallic character. The center-to-center separation of the SWCNT is ~0.7nm. The Fermi-level was adjusted to 0eV for better readability.

**Figure 3:**
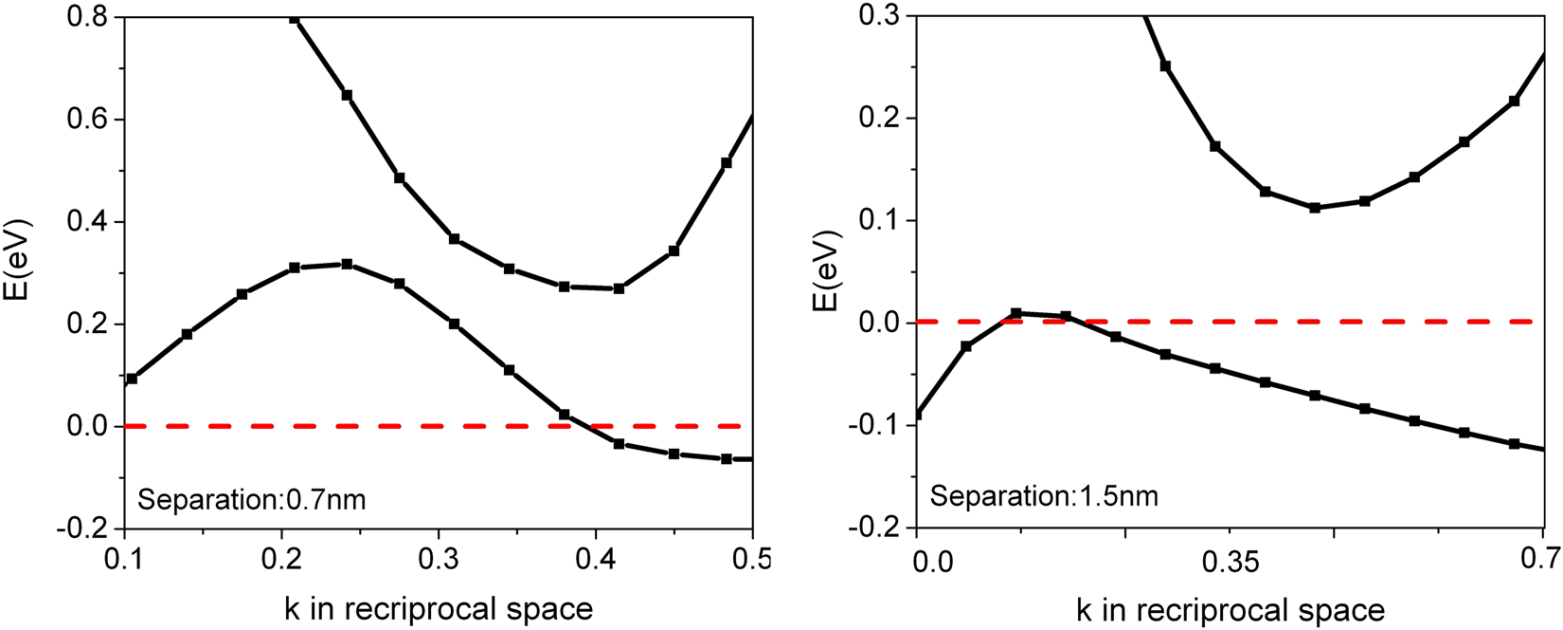
The local band structures of (4,2) SWCNTs in the center-to-center separation of ~0.7nm and ~1.5nm, respectively. The tube-to-tube interaction triggers a semiconductor to metal transition.

In experimental work on superconducting SWCNT arrays grown in AlPO_4_-5 zeolite matrices containing a mixture of nanotubes with different chiralities with mostly (4,2) and (5,0) SWCNT^5^, the measured onset *T*_c_ at 15 K is thus likely attributed to the (4,2) SWCNT, in contrast to what has been proposed previously^26^, and the sharper downturns of resistance at ~7 K at zero magnetic field^5^ is likely triggered by the onset of superconducting fluctuations in the (5,0) nanotubes, which dramatically enhances the transverse Josephson coupling within the array, and thus triggers a transition towards a three-dimensional bulk superconducting state within the array. The electronic energy band diagrams of the SWCNT using a built-in coordinate system are metallic as shown in Fig. 2. At a first glance, this contradicts to earlier work on individual nanotubes where a bandgap was found for (4,2) nanotubes^26^. However, the difference originates from the arrangement of the nanotubes within a hexagonal array with weak transverse coupling. The 0.12 eV indirect band gap of the (4,2) SWCNT in the separation of ~1.5 nm computed by “Quantum ESPRESSO” is suppressed by lateral coupling as shown in Fig. 3. The valence and conduction bands are clearly overlapped if the (4,2) SWCNTs are spaced by ~0.7 nm. The center-to-center distance of ~0.7 nm corresponds to the wall-to-wall distance of ~0.3 nm, which is still much larger than single bond length, and therefore we do not expect any extra covalent bond forms laterally across the tubes^27^. The metal-semiconductor transition point may occur at the center-to-center distance of ~1.13 nm, roughly based on the linear interpolation of two band edges as a function of tube-to-tube separations.

When graphene is rolled into the form of nanotubes, radial and tangential components of the atomic spring constants need to be considered. The out-of-plane vibration of the carbon is neglected, because there are no nearest neighbors along the radial axis. As a result, the tangential vibration is weakened by a sinusoidal factor, causing the typical phonon softening found in low dimensional materials. The slower lattice vibration allows longer electron-phonon scattering times, causing a *T*_c_ is enhancement. For instance, the *T*_c_ of 1D Pb nanowires (or Sn nanowires) is enhanced by a factor of ~1.6 compared to the bulk *T*_c_ by this surface phonon softening effect^28,29^.

**Figure 4:**
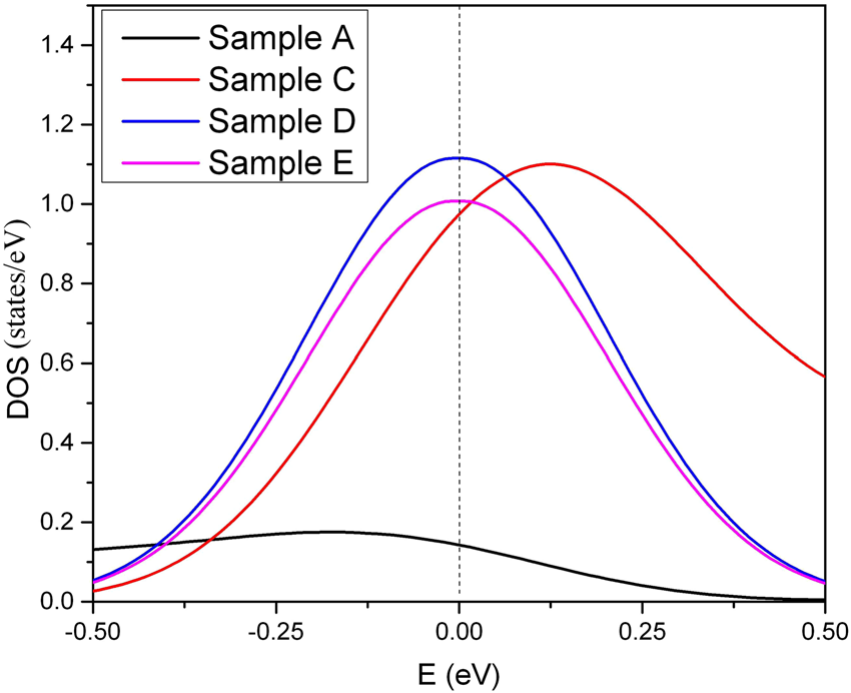
The electronic density of states (DOS) per atom in different samples. The DOS at Fermi level of Sample A is much lower than for any other sample. The DOS of the Sample B and C are assumed to be identical because of the weak global curvature.

Sample B is produced by shortening the armchair SWCNT to one repeating unit in the larger diameter of 2.3 nm that shows a remarkable effect on *T*_c_ which is increased to 52 K. The main reason is that the DOS (E_F_) of Sample B is ~10 times larger than for Sample A as displayed in Fig. 4. One might question the validity of our scale factor approach (see Methods section for details) when the SWCNT is shortened to a carbon ring. However, the Debye temperature of the carbon chain is even higher than the SWCNT^30,31^, and therefore using the scale factor to predict the *T*_c_ of the carbon nanowire is even more accurate. In the absence of phonon softening due to curvature, the electron phonon coupling is lowered^23,28,32^, and therefore unfolding the Sample B to Sample C lowers *T*_c_ by 1.5 times. Removal of the kinks leads to Sample D with a 23 K *T*_c_. This is also explained by the loss of local curvature, which weakens the electron phonon coupling. However, the curvature is not the only parameter we can tune in order to optimize *T*_c_^15,20^. Indeed, if we decrease the bond length from 143 pm to 134 pm, as demonstrated in Sample E, the predicted *T*_c_ is enhanced tremendously from 23 K to 81 K. The calculation based on the scale factor approach is illustrated in Table 4.

**Table 4:** The ${T}_{c}^{scale}$ calculation in various materials. The reference material is Al with ${\lambda }_{ref}=0.38$. The C-chains are separated by 1340 pm. The compressed lithium is shown here as an example to estimate${\lambda }_{scale}$ ^44,45^.

Material	*R*_*W*_	*R*_*DOS*_	${{\boldsymbol{R}}}_{\omega }$	*R*_*M*_	*R*_*u*_	${{\boldsymbol{R}}}_{a(q)}$	${\boldsymbol{\lambda }}/{{\boldsymbol{\lambda }}}_{ref}$	${{\boldsymbol{\lambda }}}_{scale}$	µ	${\boldsymbol{f}}({T}_{D})$	${T}_{c}^{scale}$ (K)
Al	1	1	1	1	1	1	1	0.38	0.11	1.0000	1.2
Li (35 GPa)	1.59	0.64	2.82	0.26	6.94	0.89	7.34	2.79	0.14	1.0034	11.8
Sample D	3.92	1.59	4.06	0.44	3.57	0.83	14.0	5.25	0.15	1.0022	22.6
Sample E	8.81	1.61	5.13	0.44	5.10	1.01	48.4	18.4	0.15	1.0004	81.1

**Figure 5:**
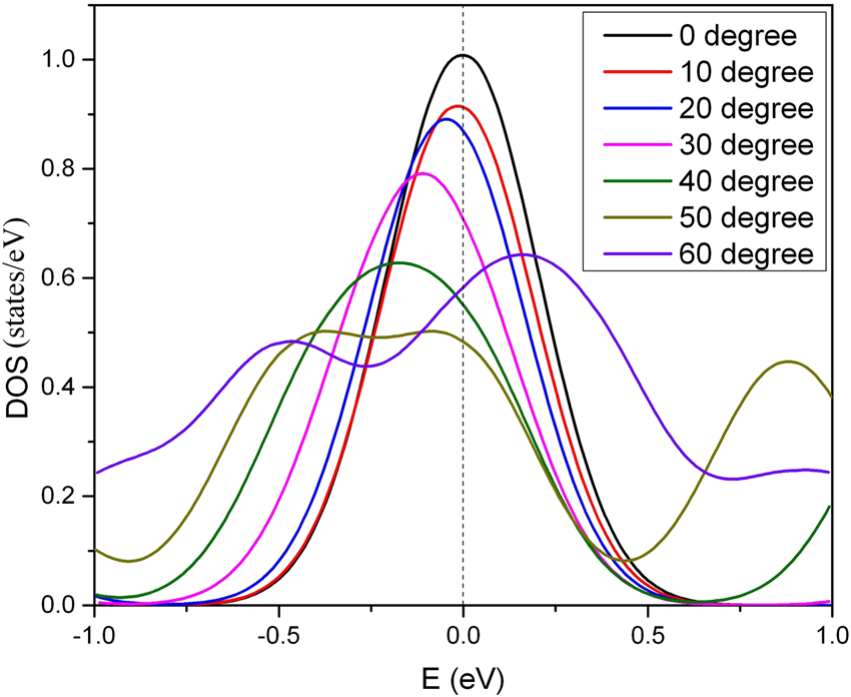
The electronic density of states (DOS) per atom in Sample F as a function of kink angle. The Fermi-level, which is adjusted to 0 eV, coincides with the van Hove singularity in the DOS at zero degrees.

**Figure 6:**
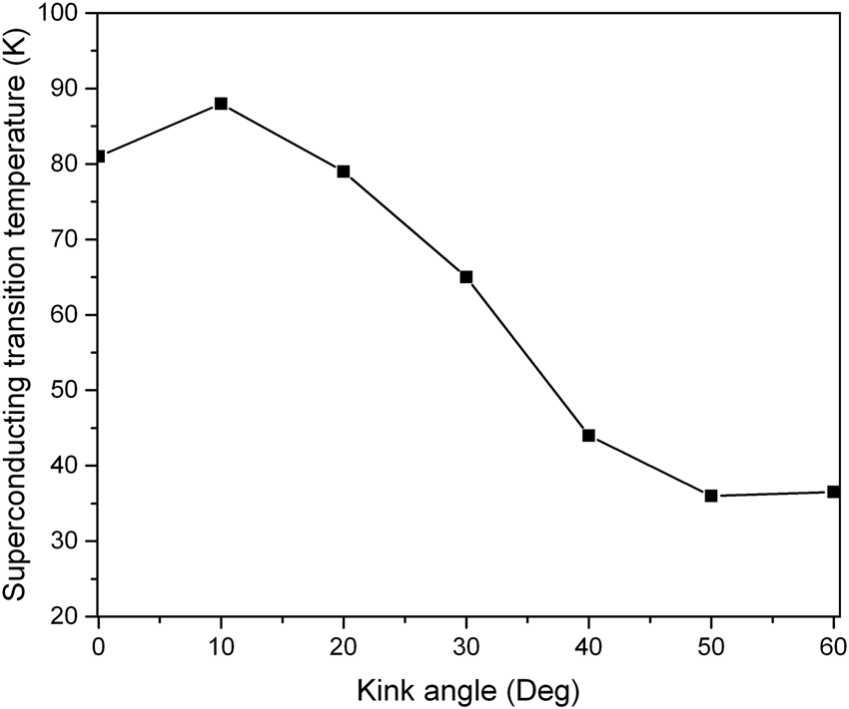
The *T*_c_ of the Sample F as a function of kink angle. The *T*_c_ is increased to 88K after the kink is bent to 10 degree. However, a dramatic reduction of *T*_c_ occurs at larger kink angles.

The ratio of branch length *R*_B_ in Sample F equals to 3. Figure 5 demonstrates that the DOS is shifted to lower energies when the kink angle is increased from 10 to 50 degrees, except for the case of 60 degrees. The sharpness of the DOS distribution decreases with increasing kink angle owing to a denser particle concentration. It stimulates us to investigate the dependence of *T*_c_ of Sample F as a function of the kink angle. We observe that *T*_c_ becomes optimized at 10 degrees (Fig. 6). Two effects are competing. On one hand, the electron phonon coupling benefits dramatically from the local curvature^28,32^. On the other hand, the electron phonon coupling is weakened by the loss of electronic density of states^33^. The effect of the local curvature is not sufficient to compensate the effect of the DOS for large kink angles, and hence *T*_c_ becomes significantly reduced beyond 10 degrees. As the carbon ring (Sample G) consists of ~100 atoms only, the tilt angle between the adjacent atoms is about 360/100 = 3.6 degrees which is ~8 times smaller than the (4,2) SWCNT. The few degrees of curvature do not amend the electronic band diagram and the electronic DOS remarkably^23^, as demonstrated in the Methods section. Modifying the lattice vibrations is sufficient to dramatically tune *T*_c_ of such weakly-curved low-dimensional carbon-based superconductors^23^. As a result, we use the electronic band diagram and the electronic DOS obtained from the linear carbon chain to model a carbon ring in the following. If Sample F is finally bent to form a ring structure of diameter of 4.2 nm (Sample G), *T*_c_ becomes optimized and reaches a value as large as 115 K. The *T*_c_ is thus enhanced by a factor of 1.3.

**Figure 7:**
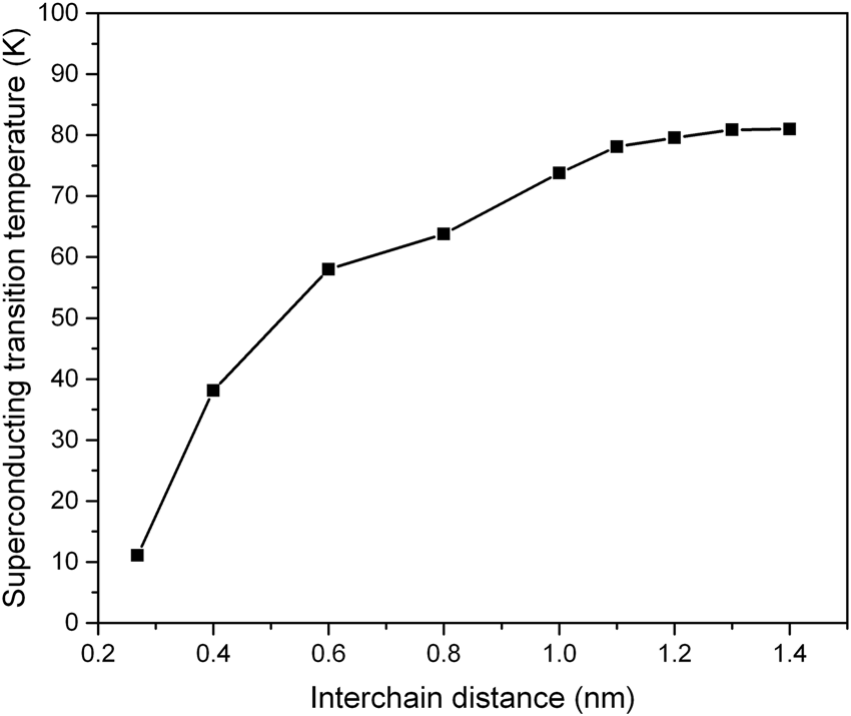
The *T*_c_ of the laterally coupled carbon chains (Sample E) as a function of chain-to-chain separation.

**Figure 8:**
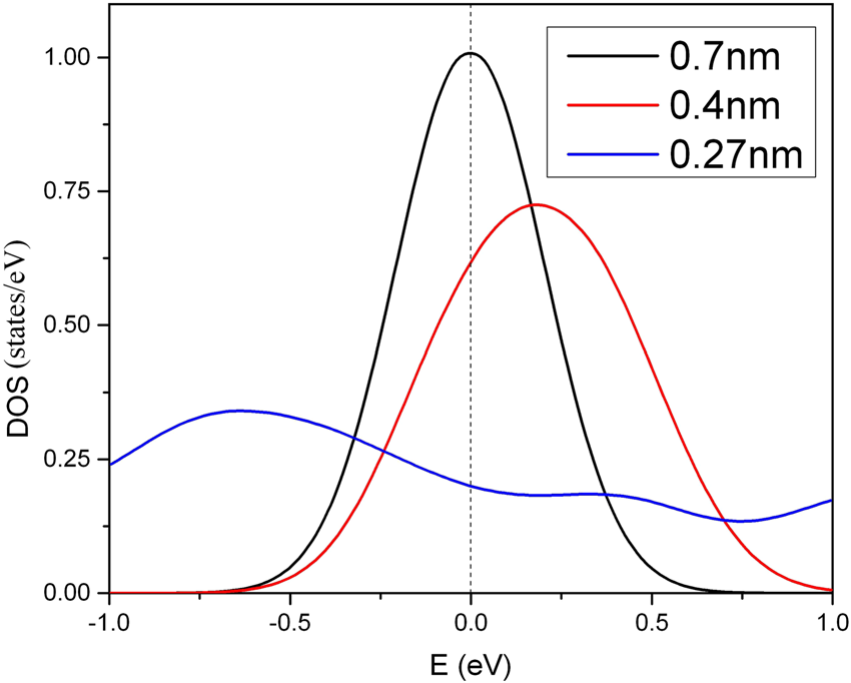
The electronic density of states of the coupled arrays of Sample E at various lateral coupling strengths. The sharpness of the DOS peaks rapidly decays at close chain density within the array.

Another interesting phenomenon is observed in the coupled linear carbon nanowires (Sample E) as shown in Fig. 7. The *T*_c_ remains constant for chain separations exceeding 1.2 nm. However the *T*_c_ is suppressed due to the lateral coupling, because the DOS(E_F_) of the coupled nanowires are significantly reduced for intra-chain distances of less than 7 nm (Fig. 8). However, there may exist an opportunity to increase *T*_c_ in the presence of strong chain-to-chain couplings in 1D chains of materials with larger atomic numbers, because the larger atomic number of the element, the stronger the Coulomb attraction the material generates.

**Figure 9:**
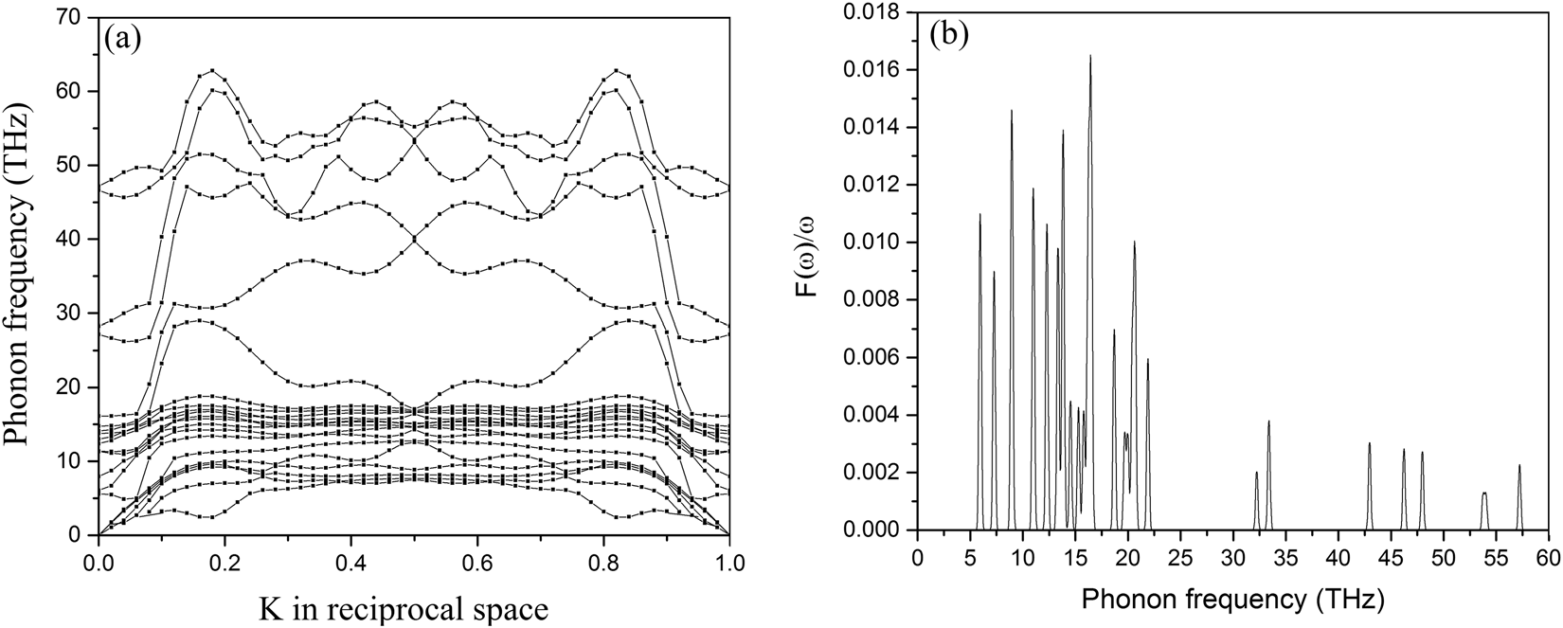
Phonon dispersion relation (**a**) and phonon spectrum (**b**) of Sample F obtained from “Quantum ESPRESSO”.

The simulation of the coupled chains is tremendously important, because the low dimensionality of the isolated kink structural carbon ring would cause strong phase slip events along the chain, and thus should cause finite resistance at all temperatures in the superconducting state^6^ unless the rings are coupled laterally via the Josephson effect^28^. According to recent theoretical and experimental work^5,8,34,35^, a dimensional crossover towards a three-dimensional bulk superconducting state in cylindrical superconducting nanowire arrays triggered by the transverse Josephson effect is possible. This dimensional transition should be unaffected by the curvature, which only influences the critical temperature where the Cooper pairs form. The coherence length $\zeta$ of the kink structural carbon ring is about 12 nm as obtained from $\zeta =\frac{\hslash {v}_{F}}{\pi \Delta }$, where ${v}_{F}$ is Fermi velocity^33^. If the carbon rings are arranged on top of each other with a ring-to-ring distance along the axial direction of 1.2 nm, a Berezinskii-Kosterlitz-Thouless-like (BKT) transition^36^ is likely able to trigger the dimensional crossover from 1D to 3D^8^, because the coherence length exceeds the ring-to-ring separation. As a result, the global phase coherence in the coupled kink structural carbon ring is expected to establish a true bulk superconducting zero resistance state at finite temperature^8,28^. However, the strength of phase fluctuations causing phase slip events will depend crucially on the Josephson coupling between the rings, and it is unclear whether there would remain a large separation between *T*_c_ (where the Cooper pairs form in the individual rings) and the temperature (*T*_BKT_) below which global phase coherence and thus zero resistance occurs. We compare the electron phonon coupling of the sample F obtained from the well accepted formula, ${\lambda }_{Mc}=2\int {\alpha }^{2}\frac{F(\omega )}{\omega }d\omega$, with help of the phonon data in Fig. 9. The Debye temperature of the sample F is ~1500 K. We only show the phonon data of sample F for clarity. The ${\alpha }^{2}$contains the average square electron-phonon matrix^24^. The ${T}_{c}^{Mc}$ estimated from the McMillian method can still reach more than 40 K, as shown in Table 5. The renormalized factor $(1+\lambda )$is valid in the strong coupling regime.

**Table 5:** The *T*_*c*_ attained from the McMillian method. The ${T}_{c}^{scale}$ is obtained from ${\lambda }_{scale}$ instead of ${\lambda }_{Mc}$.

Material	${\lambda }_{Mc}=2\int {\alpha }^{2}(F(\omega )/\omega )d\omega$	$\lambda /(1+\lambda )$	µ	${\boldsymbol{\mu }}/({\bf{1}}+{\boldsymbol{\lambda }})$	${{\boldsymbol{T}}}_{{\boldsymbol{c}}}^{{\boldsymbol{Mc}}}$ *(K)*	${T}_{c}^{scale}$ *(K)*
Sample E	1.51	0.60	0.15	0.059	41.9	81
Sample F	1.53	0.61	0.15	0.059	42.6	88

A large discrepancy between ${T}_{c}^{Mc}$ and ${T}_{c}^{scale}$ is observed in the SWCNT array. If the diameter is reduced to one atom thickness only, as in the case of a carbon chain, we expect the error to increase further. In view of this, the formation of Cooper pairs in sample E at 81 K is likely more reasonable. The average phonon frequencies of the samples are shown in Table 6. The absolute value of the phonon frequency is not important in the scale factor approach, which requires a ratio of phonon frequencies only.

**Table 6:** Phonon frequencies of the various carbon structures.

Sample	A	B	C	D	E	F	G
<ω> (THz)	36	5.5	12	21	27	26	15

Table 7 shows the reduction of *T*_c_ for large diameter rings. The smaller ring provides a higher *T*_c_ due to the curvature-induced phonon softening. All our attempts to increase the *T*_c_ beyond our predicted 115 K failed so far. For example, reducing the radius of the kink structural carbon ring would reinforce the phonon softening. However, the electron phonon coupling is weaker if the nanowire becomes too short^37^. Our 115 K ($\lambda  \sim 25$) prediction thus appears to be the optimal *T*_c_ value, although there may exist more complex carbon structure that could push this high temperature superconductor limit in carbon based structures further. In the end it will be the experimental realization of such a carbon ring array structure, which may or may not confirm our theoretical prediction.

**Table 7:** The *T*_c_ of the kink structural carbon ring for various ring diameters.

Total number of atoms in Sample G	Superconducting transition temperature/K
100	115
130	96
170	90

**Figure 10:**
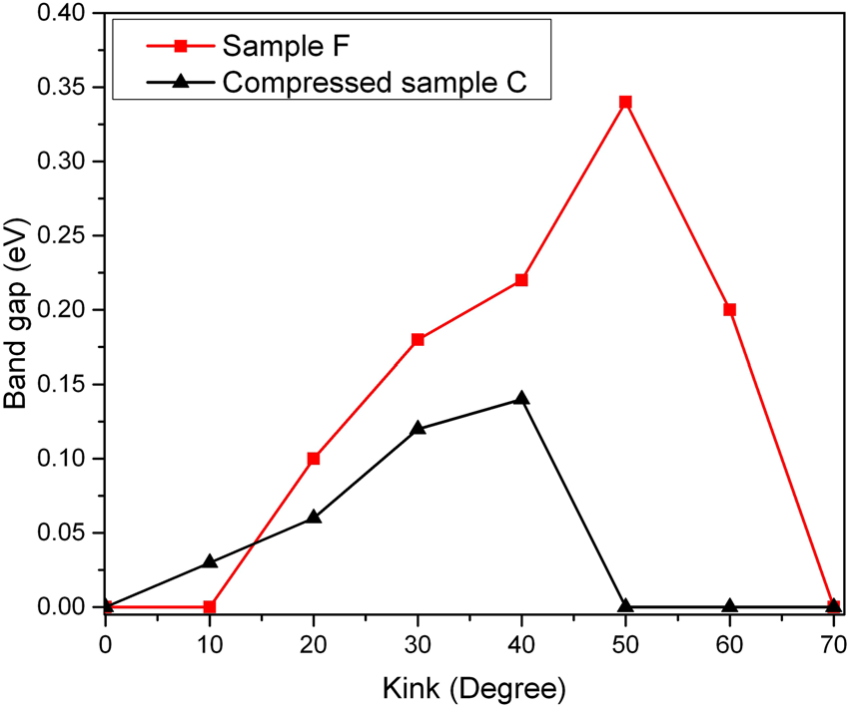
The band gap of Sample F and the “compressed” Sample C as a series of kink angles. Here the bond length of the Sample C is compressed to 134pm.

**Figure 11:**
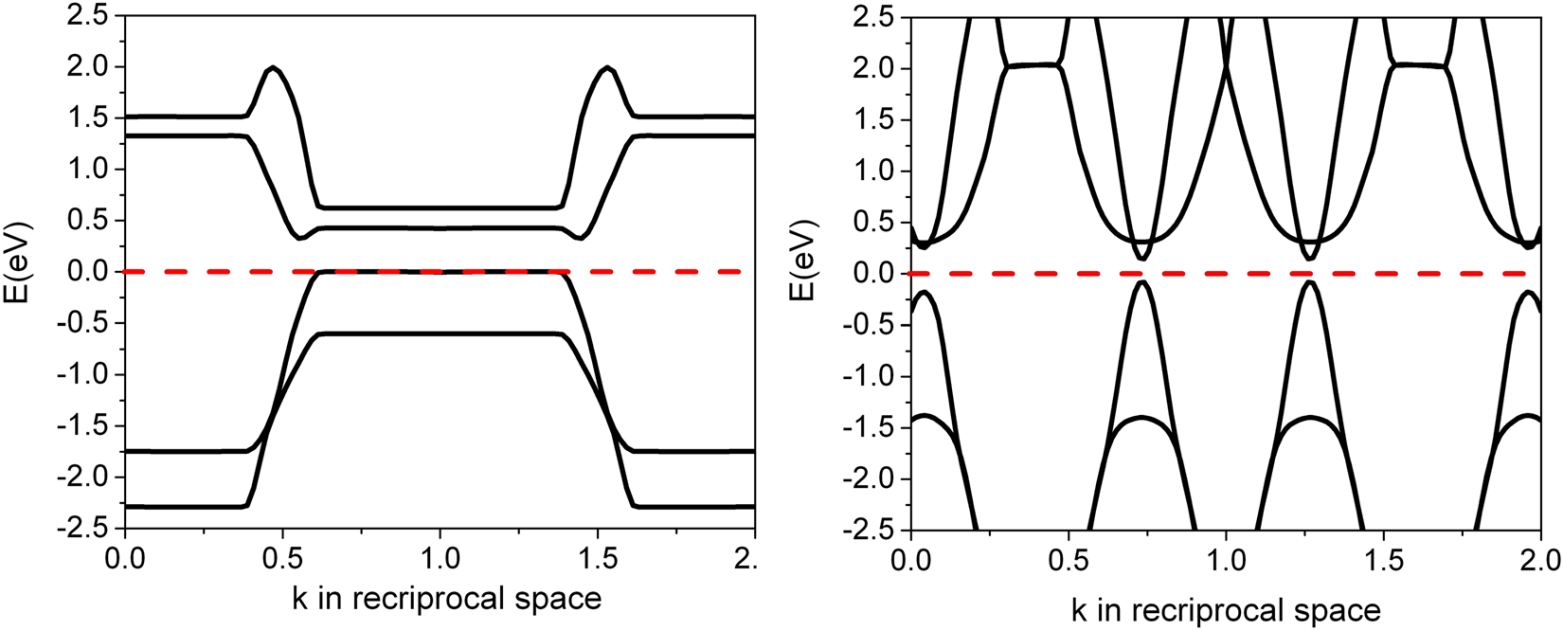
The band diagram of the Sample F at 50 degrees kink angle (left) and of the compressed Sample C at 40 degrees kink angle (right). The red lines correspond to the shifted Fermi levels.

A final issue to discuss is whether or not there would be a Peierls transition at finite temperature, which would destroy our dream of a novel carbon-based high temperature superconductor irrevocably. The linear carbon chain indeed transforms from the metallic β-carbyne (cumulene) with repeating double bonds to the semiconducting α-carbyne (polyyne) with alternating single and triple bonds at 500 K due to a Peierls-type of transition^15,20^. However, such linear carbon chains represent a very unusual case, where the Peierls distortion occurs in the high temperature phase, while below 500 K the metallic phase is stable. Although carbon nanotube and graphene show bond distances of 143 pm^5,12^, the bond length of 143 pm in the carbon nanowire is likely impossible unless it is strained. Both Monte Carlo simulation and DFT calculation provide theoretical evidence that show relaxed bond lengths of carbon nanowires of ~134 pm and ~127 pm, respectively^20,38^, even though the two simulation methods are entirely different. Hence the bond distance of the nanowire at ~130 pm is within our expectation. Needless to say, nano-devices operating at room temperature based on semiconductor junctions open tremendous opportunities in daily-life applications, and the possibility to tune the semiconducting gap of low-dimensional carbon structure in a similar manner as our approach of tailoring a high temperature carbon based superconductor appears equivalently exciting. As we will discuss in the following, it is indeed dependent on tiny details, whether or not the ground state of the materials is semi- or superconducting^15^. We attempt to modify the electronic band structure of cumulene in order to trigger the metal to semiconductor transition at 300 K. Indeed, the local curvature of the carbon nanowire can open a tiny band gap as demonstrated in Fig. 10 and Fig. 11. This phenomenon is observed similarly in ultrathin silicon nanowires in which the band gap of silicon nanowires increases with decreasing diameter^39^. The optical band gap of the kink structured carbon nanowire is unlikely due to a Peierls transition. Despite the branches regroup the collective lattice spacing from a geometrical point of view at all kink angles, the electrons may not follow quantum-mechanically, because the band gap of the carbon chains is entirely suppressed with a strong curvature. Figure 11 shows that the band gap of the carbon chain is larger if the kink density is smaller and presumably decreases the particle density. However, the minimum kink angle triggering the return of the metallic phase is influenced by the local curvature. The minimum kink angle can be smaller under the stronger local curvature in the compressed Sample C. The superconducting ground state we predicted previously is thus possible, but our discussion above also demonstrates the subtle balance between the different structural parameters (local and global curvature, bond lengths, intra-chain separation within an array) influencing the electronic properties in these low-dimensional carbon structures, and how tiny variations in this balance can lead to entirely different electronic ground states. In the end it will be the experimental realization of these structures who will entirely reveal their potential for electronic applications. Fortunately, there has been a recent breakthrough in the fabrications of individual cumulene chains^2–4^, which suggests that the experimental realization of single crystalline bulk samples may be possible in the near future.

## Methods

The pairing Hamiltonian, ${H}_{pair}=\sum _{k\sigma }{E}_{k}{n}_{k\sigma }+\sum _{kl}{V}_{kl}{c}_{k\uparrow }^{\ast }{c}_{k\downarrow }^{\ast }{c}_{l\uparrow }{c}_{l\downarrow }$, is composed of the single particle energy *E*_*k*_ relative to the Fermi energy and the interaction term ${V}_{kl}$, which scatters the particle from a state with $(l\uparrow ,-l\downarrow )$ to $(k\uparrow ,-k\downarrow )$. The creation operators, ${c}_{k\uparrow }^{\ast }$and ${c}_{k\downarrow }^{\ast }$, correspond to spin up and down, respectively, while ${n}_{k\sigma }$is the particle number operator, $\sigma$is the spin index^33^. We define ${\psi }_{G}$ to be the ground state of the BCS wavefunction

1 $$|{\psi }_{G}\rangle =\prod _{k={k}_{1}\mathrm{....}{k}_{M}}({u}_{k}+{v}_{k}{c}_{k\uparrow }^{\ast }{c}_{k\downarrow }^{\ast })|{\phi }_{0}\rangle $$

where $|{\phi }_{0}\rangle$ is the vacuum state with no particles present and ${|{u}_{k}|}^{2}$ refers to the unoccupied probability since ${|{u}_{k}|}^{2}+{|{v}_{k}|}^{2}=1$. The energy gap Δdoes not depend on *k* and hence we may write $\Delta ={\Delta }_{k}=-\sum _{l}{V}_{kl}{u}_{l}{v}_{l}$ ^33^. Multiplying $-\sum _{k}{u}_{k}{v}_{k}$to both sides of the above equation leads to

2 $$-\Delta \sum _{k}{u}_{k}{v}_{k}=\sum _{kl}{V}_{kl}{u}_{k}{v}_{k}{u}_{l}{v}_{l}$$

The energy gap can then be expressed as $\Delta =\frac{{H}_{e-ph}}{-\sum _{k}{u}_{k}{v}_{k}}$. If the ratio of $-\sum _{k}{u}_{k}{v}_{k}$(i.e. the division of transfer function) between two superconductive materials is close to 1, $\frac{{\Delta }_{A}}{{\Delta }_{B}} \sim \frac{{H}_{e-ph(A)}}{{H}_{e-ph(B)}}$ is satisfied at 0 K. The interaction term arises from the electron phonon coupling in the expression of ^23,33^

3 $${H}_{e-ph}=\sum _{kk\text{'}\sigma \lambda }\{[(\int {d}^{3}r{\varphi }_{k\text{'}\sigma }^{\ast }({\bf{r}}){\varphi }_{k\sigma }({\bf{r}}){\nabla }_{{R}_{i}^{0}}V({\bf{r}}-{{\bf{R}}}_{i}^{0}))\cdot {e}^{\lambda }({\bf{q}})]\sqrt{\frac{N}{2M\omega }}{c}_{k\text{'}\sigma }^{\dagger }{c}_{k\sigma }(({a}_{\lambda }({\bf{q}})+{a}_{\lambda }^{\dagger }(-{\bf{q}}))\}$$

4 $$\sum {c}_{k\text{'}\sigma }^{\dagger }{c}_{k\sigma }={n}^{ion}(1-\frac{1}{\varepsilon })={n}_{T}\nabla \cdot u$$

where ${n}^{ion}$ are the ionic charge fluctuations induced by ionic displacement and ${n}_{T}$ is the total ionic charge density. The polarization vector $\sum _{\alpha }{{e}_{\alpha }}^{\lambda }(k)\cdot {{e}_{\alpha }}^{\lambda \text{'}}(k)={\delta }_{\lambda \lambda \text{'}}$ is included in the orthogonal cases. $V(r)$ is the lattice potential as a function of special position. The electric fields emitted from the face centers, body center or kink structure in different materials is resolved into orthogonal directions before solving the Schrodinger equation of the electrons. We abbreviate$\int {d}^{3}r{\varphi }_{k\text{'}\sigma }^{\ast }({\bf{r}}){\varphi }_{k\sigma }({\bf{r}}){\nabla }_{{R}_{i}^{0}}V({\bf{r}}-{{\bf{R}}}_{i}^{0})\cdot {e}^{\lambda }({\bf{q}})\sqrt{\frac{N}{2M\omega }}$ as ${g}_{kk\text{'}}$. ${{\bf{R}}}_{i}^{0}$ is the equilibrium position of the i^th^ atom and $M$is the atomic mass. The dielectric factor $\varepsilon$serves to consider the screening effect. The ${a}_{q\lambda }^{\dagger }{a}_{q\lambda }+\frac{1}{2}$ is defined as the quantum number of the phonons, where ${\bf{q}}={\bf{k}}-{\bf{k}}\text{'}+{\bf{G}}$ and **G** corresponds to a reciprocal lattice vector^33^. The ionic displacement is $u=\frac{1}{\sqrt{NM}}\sum _{k\lambda }{Q}_{\lambda }{e}^{\lambda }{e}^{ik\cdot {R}_{i}^{0}}$. Finally, we obtain ${a}_{\lambda }({\bf{q}})+{a}_{\lambda }^{\dagger }(-{\bf{q}})$ according to ${a}_{\lambda }({\bf{q}})+{a}_{\lambda }^{\dagger }(-{\bf{q}})={Q}_{\lambda }\sqrt{2{\omega }_{\lambda }}$.

The electronic band diagram, dispersion relationship of phonons and the density of states of electrons and phonons are calculated by the GGA functional (CASTEP)^21,22^ in Materials Studio 7 (unless otherwise specified). The energy cut-off point and tolerance are 200 eV and 10 µeV, respectively. The 3D time-independent Schrödinger equation of electrons is solved by the method of separation of variables^33^. The potential energy *U* in each direction is summed up to 100 unit cells in the Schrödinger equation. The percentage change in the $\frac{\sum _{1}^{100}U-\sum _{1}^{99}U}{\sum _{1}^{100}U}$ is about 0.1% and therefore it can be regarded as an infinitely large system. In other word, the size effect on the electronic band structure and density of states is negligible. For circular materials, the attractive force acting on the electrons is modified due to the change of the effective atomic number ${Z}_{effective}$ ^40^.

5 $${Z}_{effective}=Z\frac{\sum _{r}^{R}{U}_{c}(r)}{\sum _{r}^{R}{U}_{p}(r)}$$

The Bloch theorem states that the wavefunctions of electron $\psi$ has the form $\psi (r+R)={e}^{ik\cdot R}\psi (r)$ where *k* is the wave number and *R* is a lattice vector. The ${Z}_{effective}$ is estimated by comparing the attractive potential between the circular ${U}_{c}$ and planar ${U}_{p}$ shapes. The computation of the phonon is based on the algorithm of finite displacement in the CASTEP. The supercell cut-off radius is 0.5 nm. The wavefunction of phonon is solved with help of Hermit polynomial^41^. Due to the low temperatures, considering the lowest order in the Hermit polynomial is a sufficiently precise approximation.

The prediction of the *T*_c_ is acquired by computing the scale factor $\frac{{T}_{c(P)}}{{T}_{c(Q)}}=\frac{{\Delta }_{P}(0)}{{\Delta }_{Q}(0)}$, because $\Delta (0)\propto {T}_{c}$ per electron^33^. If the *T*_c_ of the material *Q* is known, the *T*_c_ of the material *P* can be predicted according to the above *kl* dependent scale factor. The scale factor is used instead of calculating the *T*_c_ directly, because the phonon wavefunction is solved in dimensionless units. However, the ${u}_{l}{v}_{l}$ depend on Δ and hence another transfer function is required: According to BCS theory, the energy gap can be estimated via ${\Delta }_{k}=-\frac{1}{2}\sum {V}_{kl}\frac{{\Delta }_{l}}{{({\Delta }_{l}^{2}+{E}_{k}^{2})}^{0.5}}$ ^33^. The transfer function is derived by calculating the trial energy gap ${\Delta }^{T}$, which originates from the electrons at the Fermi level only. In this circumstance the ${\Delta }^{T}$ is directly proportional to the interaction term since ${E}_{k}=0$. Then the interpreted transfer function, i.e. ${u}_{l}^{T}{v}_{l}^{T}$as a function of electron energy, will be substituted into ${\Delta }_{k}^{corrected}=-\sum _{kl}{V}_{kl}{u}_{l}^{T}{v}_{l}^{T}$ in order to correct the energy gap. However, the limitation of using the transfer function is to satisfy the condition for the Debye energy $\hslash {\omega }_{D}\gg \Delta$. Otherwise, the BCS occupational fraction will not drop sharply to zero when the electron energy increases^33^.

Before we implement the *T*_c_ of the kink structural carbon nanowire, we have checked carefully that the scale factor does not depend on the lattice structure and curvature. We present the accuracy of the scale factor approach as shown in Tables 8 & 9. We define ${T}_{c}^{P}={T}_{c}^{Q}|\frac{{\lambda }_{P}-{\mu }_{P}}{{\lambda }_{Q}-{\mu }_{Q}}\frac{1}{f({T}_{D})}|$, where $f({T}_{D})$is the ratio of the transfer function between two materials P and Q, ${T}_{D}$ is the Debye temperature. $\lambda$is the electron phonon scattering term, *μ* is the Coulomb screening. ${T}_{c}^{ex}$and ${T}_{c}^{Mc}$ are the ${T}_{c}$values obtained from experimental data and the McMillian formula, respectively. The ${T}_{c}^{scale}$ values in Table 9 are obtained by modifying the${g}_{kk\text{'}}$ only.

**Table 8:** The *T*_*c*_calculation based on the scale factor approach^25^.

Materials	µ	λ	${\boldsymbol{f}}({T}_{D})$	${T}_{c}^{ex}$ (K)	${{\boldsymbol{T}}}_{c}^{scale}$ (K)	${T}_{c}^{Mc}$ (K)
Al	0.11	0.38	1.0000	1.2	1.2 (Reference)	2.4
Pb	0.10	1.55	1.0227	7.2	6.3	7.0
Sn	0.10	0.72	1.0102	3.7	2.8	5.1
In	0.10	0.69	1.0108	3.4	2.4	3.1
Hg	0.10	1.00	1.0224	4.2	3.9	4.1
Ta	0.10	0.87	1.0101	4.4	3.4	5.8
Li (35 GPa)	0.14	2.75	1.0034	10.0	11.6	13.0
Mo	0.11	0.32	0.9280	0.9	1.0	0.3
Ga	0.11	2.25	1.0157	8.6	9.3	14.1

**Table 9:** The estimated *T*_*c*_ values in various low dimensional superconductors based on the scale factor approach and the empirical method^28,29,32,46^.

Material	${{\boldsymbol{\lambda }}}_{scale}$	µ	${{\boldsymbol{R}}}_{shape}$	${T}_{c}^{scale}$ (K)	${T}_{c}^{ex}$ (K)	*Scaled by*
Al (2D)	0.43	0.12	1.1	1.3	1.3	Al (3D)
Sn (2D)	0.79	0.11	1.1	3.9	4.0	Sn (3D)
Sn(1D)	0.92	0.10	1.3	4.9	5.5	Sn (3D)
Pb (2D hollow sphere)	2.37	0.10	1.5	11.2	11.0	Pb (3D)
Pb (1D)	2.10	0.10	1.4	10.0	11.3	Pb (3D)

**Figure 12:**
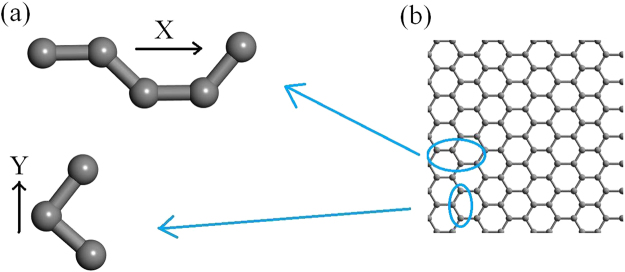
(**a**) Two types of repeating units of graphene. (**b**) The structure of graphene.

In the next step we apply this method to calculate the *T*_c_ of arrays of 4 Ångstrom SWCNT, which are formed from hexagonal structure in the presence of curvature. A SWCNT can be produced by rolling graphene into the shape of a tube. The phonon wavefunction in a SWCNT $\chi ({\omega }_{x}^{planar},{\omega }_{y}^{planar},{\omega }_{z}^{circular})$ is going to connect with the graphene $\chi ({\omega }_{x}^{planar},{\omega }_{y}^{planar})$ and linear carbon nanowire $\chi ({\omega }_{x}^{linear})$ in order to minimize computational cost. The spring constants in the graphene are split into two types of repeating units as shown in Fig. 12. The effective spring constants $({K}_{x}^{planar},{K}_{y}^{planar})$ of the upper and lower repeating units are resolved into the *x* and *y* axis, respectively.

As the bond length of graphene is about 143 pm, we make use of the GGA functional^21,22^ in the CASTEP to simulate the dispersion curve and the phonon density of states in the linear carbon chain to interpret the vibrational frequency, considering the same bond distance of 143 pm. The four nearest carbon atoms along the reference carbon nanowire are grouped to form a linear repeating unit with the known dimensionless spring constant ${K}_{x}^{linear}$. The ratio of the resultant spring constant of the graphene relative to the reference chain can be computed by $\frac{\sqrt{({K}_{x}^{planar}{K}_{x}^{planar}+{K}_{y}^{planar}{K}_{y}^{planar})}}{{K}_{x}^{linear}}$. Based on classical mass-spring approach, the vibrational frequency of the graphene is presumably interpretable and therefore the approximated phonon wavefunction in graphene can be estimated^41^. The approximated wavefunction of the SWCNT is determined in combination with the concept of surface phonon softening due to the curvature. About 13 atoms along the armchair path (4,2) form a loop in the SWCNT, which means that the tilt angle between the adjacent atoms is 360/13 = 27.6 degrees. Another relative spring constant ${K}_{z}^{circular}$ of the SWCNT can be found by resolving the vector into radial and tangential components^23^ and eventually the approximated wavefunction of the SWCNT can be interpreted. As a results, ${R}_{shape}$ and ${R}_{kink}$ can be found where ${\omega }_{p}$ is not obtained from the phonon dispersion. The electronic band diagram and the electronic density of states of the SWCNT are also computed by the GGA functional in the CASTEP without making extra connection to the graphene and carbon nanowire.

The potential energy in the Schrodinger equation of electrons is found by projecting the electric fields onto the orthogonal directions again^41^. The (5,0) SWCNT is managed in a similar manner. Finally, the theoretical *T*_c_ values of the (4,2) and (5,0) SWCNT arrays can be determined.

The main interest in this article is to predict the *T*_c_ of various cumulene carbon chains with different structural arrangements in the form of kinks. The electronic density of states of the carbon chain is simulated by the GGA functional in the Dmol^3^ package^42,43^. Following the above technique to resolve the electric fields coupling to the electrons in the kink structural carbon chain, the solution of the time-independent Schrodinger equation of electrons can be determined. We will draw a parallel between the kink structural carbon chain and linear carbon chain with the same bond distance in order to determine the phonon wavefunction in the kink-structural carbon chain and the *T*_c_ as well. We will make use of the surface phonon softening^32^ to estimate the *T*_c_ when it is returned to circular shape. The predicted *T*_c_ of the Sample B, C, D, E, F and G will be obtained by utilizing a scale factor of Al, Ta, Hg, Mo, Ga, Pb, In, Sn or the (4,2) SWCNT respectively, to ensure all predicted *T*_c_ values are almost identical, despite it is scaled by different materials, different lattice structures and different curvatures. The isolated carbon chain refers to the lateral chain-to-chain separation of 1340 pm. We do not substitute the physical quantities into the conclusive equation of BCS theory directly^33^, i.e. $\Delta =\frac{\hslash {\omega }_{D}}{\sinh [1/DOS({E}_{F})V]}$, because we suspect that this conclusive formula may not work in some 1D materials with high Debye frequency. The formula is obtained by considering the finite integral of the electron energy up to the Debye frequency. If the 1D material contains extremely narrow peaks in the DOS, the difference between the DOS(*E*_F_) and DOS(*E*_F_ + d*E*) may become very large, and hence the formula likely becomes inapplicable. As a test, the prediction of *T*_c_ in the SWCNT is still accurate if we start from the original BCS pairing Hamiltonian without taking the mentioned approximations. The accuracy of the transfer function is acceptable for Tantalum and SWCNTs, because the Debye temperature is much higher than the superconducting transition temperature. If the Debye energy $\hslash {\omega }_{D}$is infinitely large, the BCS occupational probability will drop quickly to zero beyond the Fermi level, which more or less matches the pattern of the transfer function. Assuming that the ${\Delta }^{T}$ of the (4,2) SWCNT is three times larger at the same Debye frequency, the average offset^33^ between $\langle \sum _{{E}_{F}}^{{E}_{F}+3{\Delta }^{T}}{u}_{l}{v}_{l}\rangle$ and $\langle \sum _{{E}_{F}}^{{E}_{F}+{\Delta }^{T}}{u}_{l}{v}_{l}\rangle$is still less than 4% when we sum over the survival energies of the paired electrons. The accuracy of the ${\Delta }^{T}$ may not perfect, but the error of the transfer function should be significantly decreased once the Debye frequency is very large. In Table 10 we provide an overview on the important formulae we use in the scale factor approach.

**Table 10:** Useful formulae in the scale factor approach.

${W}_{kk\text{'}}=\int {d}^{3}r{\varphi }_{k\text{'}\sigma }^{\ast }({\bf{r}}){\varphi }_{k\sigma }({\bf{r}}){\nabla }_{{R}_{i}^{0}}V({\bf{r}}-{{\bf{R}}}_{i}^{0}))$ (6)	${g}_{kk\text{'}}=({W}_{kk\text{'}}\cdot {e}^{\lambda }({\bf{q}}))\sqrt{\frac{N}{2M\omega }}$ (11)
${R}_{W}=\frac{ < {W}_{kk\text{'}\lambda }^{A} > }{ < {W}_{kk\text{'}\lambda }^{B} > }$ (7)	${R}_{u}=\frac{ < \nabla \cdot {u}^{A} > }{ < \nabla \cdot {u}^{B} > }$ (12)
${R}_{a(q)}=\frac{{ < {a}_{\lambda }({\bf{q}})+{a}_{\lambda }^{\dagger }(-{\bf{q}}) > |}_{A}}{ < {{a}_{\lambda }({\bf{q}})+{a}_{\lambda }^{\dagger }(-{\bf{q}}) > |}_{B}}$ (8)	${R}_{\omega }=\frac{ < {\omega }_{A} > }{ < {\omega }_{B} > }$ (13)
${R}_{DOS}=\frac{ < DO{S}_{A}({E}_{F}\to {E}_{Debye}) > }{ < DO{S}_{B}({E}_{F}\to {E}_{Debye}) > }$ (9)	${R}_{M}=\frac{{M}_{A}}{{M}_{B}}$ (14)
${R}_{shape}=\frac{{g}_{kk\text{'}}^{A}({\omega }_{p})}{{g}_{kk\text{'}}^{B}({\omega }_{p})}$ (10)	${R}_{kink}=\frac{{g}_{kk\text{'}}^{A}({\omega }_{p})}{{g}_{kk\text{'}}^{B}({\omega }_{p})}$ (Kink ⇔ change shape) (15)
